# Bacterial Compound *N*,*N*-Dimethylhexadecylamine Modulates Expression of Iron Deficiency and Defense Response Genes in *Medicago truncatula* Independently of the Jasmonic Acid Pathway

**DOI:** 10.3390/plants9050624

**Published:** 2020-05-14

**Authors:** Vicente Montejano-Ramírez, Ernesto García-Pineda, Eduardo Valencia-Cantero

**Affiliations:** Instituto de Investigaciones Químico Biológicas, Universidad Michoacana de San Nicolás de Hidalgo, Edifico B3, Ciudad Universitaria, Morelia 58030, Mexico; piscesrhapsody@gmail.com (V.M.-R.); egpineda@umich.mx (E.G.-P.)

**Keywords:** bacterial organic volatile compound, salicylic acid, iron deprivation, cross-talk in stress-response pathways

## Abstract

Plants face a variety of biotic and abiotic stresses including attack by microbial phytopathogens and nutrient deficiencies. Some bacterial volatile organic compounds (VOCs) activate defense and iron-deficiency responses in plants. To establish a relationship between defense and iron deficiency through VOCs, we identified key genes in the defense and iron-deprivation responses of the legume model *Medicago truncatula* and evaluated the effect of the rhizobacterial VOC *N*,*N*-dimethylhexadecylamine (DMHDA) on the gene expression in these pathways by RT-qPCR. DMHDA increased *M. truncatula* growth 1.5-fold under both iron-sufficient and iron-deficient conditions compared with untreated plants, whereas salicylic acid and jasmonic acid decreased growth. Iron-deficiency induced iron uptake and defense gene expression. Moreover, the effect was greater in combination with DMHDA. Salicylic acid, *Pseudomonas syringae*, jasmonic acid, and *Botrytis cinerea* had inhibitory effects on growth and iron response gene expression but activated defense genes. Taken together, our results showed that the VOC DMHDA activates defense and iron-deprivation pathways while inducing a growth promoting effect unlike conventional phytohormones, highlighting that DMHDA does not mimic jasmonic acid but induces an alternative pathway. This is a novel aspect in the complex interactions between biotic and abiotic stresses.

## 1. Introduction

Plants are sessile organisms that interact with biotic and abiotic factors. Biotic factors include both beneficial microorganisms, such as plant growth promoting rhizobacteria (PGPR) and phytopathogens, and herbivorous insects. Therefore, plants are vulnerable to various biotic attacks [1]. To cope with biotic stress, plants have a well-developed immune system. Through the presence of membrane proteins known as pattern recognition receptors, plants can recognize diverse elicitors, such as molecular patterns associated with microbes. These elicitors include flagellin, lipopolysaccharides, peptidoglycan, elongation factors, and siderophores, which are present in beneficial microorganisms and pathogens [2]; molecular patterns associated with herbivores, which include saliva and regurgitants of herbivorous insects [3]; and molecular patterns associated with damage, which include DNA, extracellular ATP, systemin, and oligogalacturonides produced in response to damage in plant cells [4].

Once elicitors have been recognized, plants activate pattern-triggered immunity (PTI) as a first basal defense mechanism [5]. In addition to PTI, plants have systemic defense mechanisms that protect organs not exposed to microorganisms [6]. These are known as systemic acquired resistance (SAR) and induced systemic resistance (ISR) [7,8]. SAR is activated in response to pathogenic microorganisms with a biotrophic lifestyle and depends mainly on the production of salicylic acid (SA) [9,10]. ISR is activated in response to necrotrophic pathogens, herbivores, and even PGPR by increased synthesis of jasmonic acid (JA) [10,11].

Plants also interact with abiotic factors, mainly through nutrient provision, which includes iron, an essential metal for vital metabolic processes in plants [12]. Although iron is abundant, its bioavailability is restricted in alkaline or calcareous soils because it forms insoluble Fe^3+^ oxyhydroxide complexes, which are not available for plant uptake [13,14]. Therefore, plants have developed two strategies to increase iron uptake. Strategy I is used by dicotyledonous and monocotyledonous nongraminaceous plants and is based on rhizosphere acidification through proton release by an ATPase [15], followed by the reduction of Fe^3+^ to Fe^2+^ by the membrane protein ferric-chelate reductase. This is encoded by the ferric reduction oxidase 2 gene (*FRO2*) [16] and the subsequent internalization of Fe^2+^ to plant root epidermal cells by the protein iron-regulated transporter 1 (IRT1) [17]. The expression of *FRO2* and *IRT1* genes is regulated by the basic helix-loop-helix (bHLH) transcription factor FER-LIKE IRON DEFICIENCY-INDUCED TRANSCRIPTION FACTOR (FIT1) [18]. Additionally, FIT forms heterodimers with bHLH38 and bHLH39 [19], and bHLH100 and bHLH101 [20]. Strategy II is used by monocotyledonous grass plants and consists of the production and release of phytosiderophores that chelate iron to internalize the Fe^3+^ phytosiderophore complex through the protein yellow stripe 1 (YS1) [14].

Previous studies have related SAR and ISR with iron-deficiency response. In strategy I, Fe deficiency promotes the release of phenolic compounds through the protein pleiotropic drug resistance 9 (PDR9) to chelate and solubilize Fe^3+^ [21]. Additionally, these compounds exhibit both antimicrobial and antifungal activities [22]. At the genetic level, *Arabidopsis thaliana* plants grown under iron-deficient conditions show an increase in the expression of the *pathogenesis related 1* (*PR1*) and *plant defensin 1.2* (*PDF1.2*) genes, which are markers of the SAR and ISR pathways, respectively. Furthermore, when these plants were inoculated with *Botrytis cinerea*, there was a synergistic effect [23]. Similarly, there are pathogens capable of activating the response to iron deficiency, such as *Dickeya dadantii*, which, when inoculated in *A. thaliana*, increase the expression of the genes “*Natural Resistance-Associated Macrophage Protein 3*” (*NRAMP3*), *IRT1,* and *FRO2* [24].

PGPR also increase resistance to iron-deficiency stress, such as in the case of *Paenibacillus polymyxa* BFKC01, which, when inoculated in *A. thaliana,* induces the expression of *FIT1*, *FRO2,* and *IRT1* genes [25]. Similarly, these PGPR increase the expression of the *PR1*, *PR2,* and *PDF1.2* defense genes. Another beneficial microorganism that can activate both iron-deficiency response and defense pathways is *Arthrobacter agilis* UMCV2, which, in addition to promoting the growth of *Medicago sativa* [26], increases the expression of *MtFRO1*, *MtFRO2*, *MtFRO3*, *MtFRO4*, *MtFRO5,* and *MtDef2*.1 genes in *M. truncatula* plants, and the effect was synergistic when plants were grown under iron-deficient conditions [27].

Some microorganisms may activate iron-deficiency response and defense pathways through the emission of volatile organic compounds (VOCs) [28]. Treatment of *A. thaliana* plants with VOCs from the fungi *Trichoderma asperellum* and *T. harzianum* increases the expression of *bHLH38*, *bHLH39*, *FRO2,* and *IRT1* genes, which are involved in iron uptake, in addition to increasing the expression of the *PDF1.2* gene of the ISR pathway, and therefore, *A. thaliana* resistance to the fungus *B. cinerea*. The effect is similar in *Solanum lycopersicum* plants.

PGPR, such as *A. agilis* UMCV2 [26], *Sinorhizobium meliloti* 1021 [29], or *Pseudomonas fluorescens* UM270 [30], produce VOCs that induce plant growth. Of the VOCs profiled for these bacteria, *N*,*N*-dimethylhexadecylamine (DMHDA) has been highlighted. DMHDA promotes plant growth [26,31,32] and activates responses to iron deficiency through, e.g., the acidification of the rhizosphere in *M. truncatula* [31] and the induction of *SbFRO1* genes in *Sorghum bicolor* [32]. Additionally, DMHDA activates the expression of the *LOX2* gene that participates in the synthesis of JA in *A. thaliana* [33].

Recently, our research group has shown that VOCs produced by *A. agilis* UMCV2 increase the expression of *SbIRT1* and *SbIRT2* genes involved in iron transport, of *SbCOI1* involved in the ISR pathways, and *SbPR1* involved in SAR pathways [34]. However, whether DMHDA is responsible for inducing these genes remains unclear. Thus, we hypothesized that DMHDA triggers the JA pathway in plants, and through this mechanism, iron-deficiency and defense responses are induced. In the present study, we identified key genes in the defense and iron-deprivation response of the legume model *M. truncatula* and evaluated the effect of DMHDA on the expression of genes involved in the iron-deficiency and defense pathways of *M. truncatula* plants exploring the interaction between biotic and abiotic stresses.

## 2. Results

### 2.1. Effect of Iron Deficiency on M. truncatula Growth

**Figure 1:**
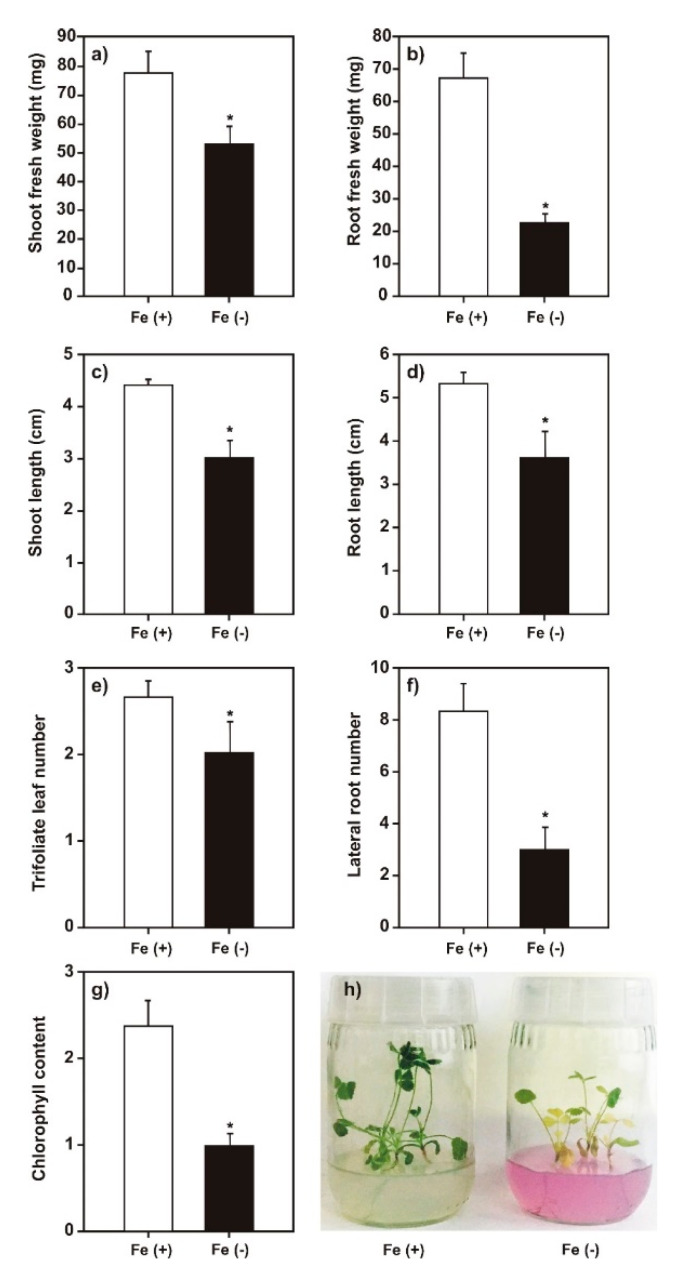
Effect of iron deprivation on *Medicago truncatula* growth. The *M. truncatula* plants cultured under iron sufficiency (control) or iron deficiency for 14 days. (**a**) Shoot fresh weight; (**b**) root fresh weight; (**c**) shoot length; (**d**) root length; (**e**) trifoliate leaf number; (**f**) lateral root number; and (**g**) chlorophyll content. Panel (**h**) show the phenotypes of plants in the treatments. The asterisk above the standard error bars indicates a significant difference between treatments calculated using Student’s *t*-test (*p* < 0.05; *n* = 9).

We employed iron-deficient growth conditions to induce iron-deficiency stress in *M. truncatula* plants. To verify the system utility, first, we analyzed the phenotype caused by our plant growth conditions. It was observed that the iron-deficient conditions decreased the length and weight of plant roots and shoots, the number of lateral roots and trifoliate leaves, and chlorophyll content compared with those of the control (Figure 1). Based on these results, we concluded that the system had effectively induced iron-deprivation stress in the plants [35].

### 2.2. Effect of SA, JA, and DMHDA on M. truncatula Growth

**Figure 2:**
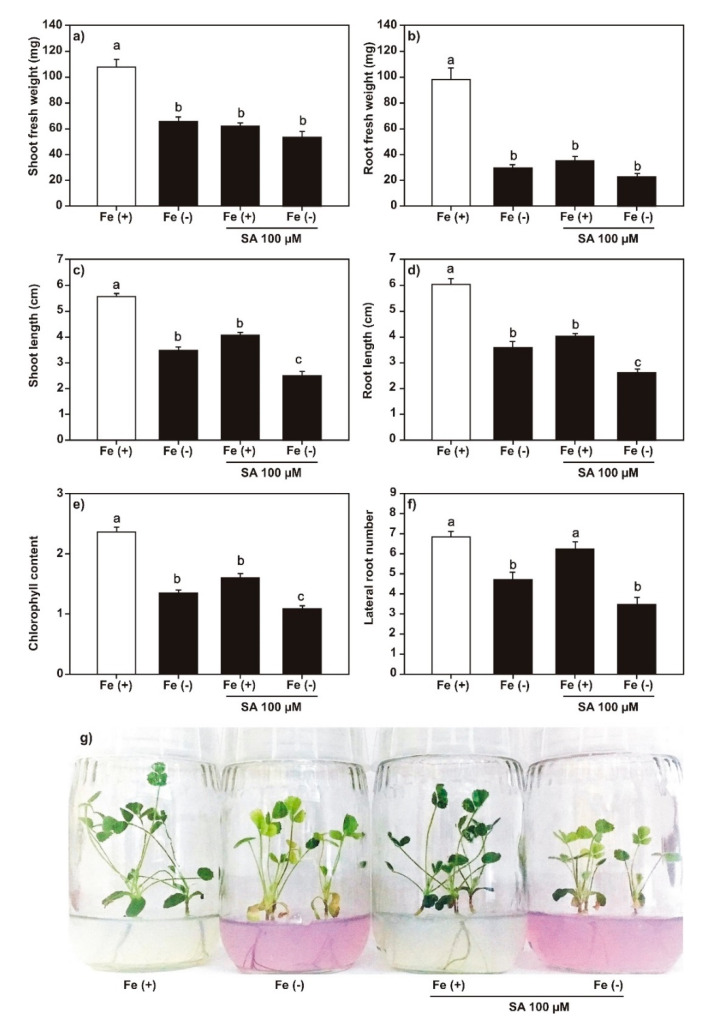
Effect of salicylic acid (SA) on *Medicago truncatula* growth. The *M. truncatula* plants were cultured in MS medium with SA (100 µM) under both iron sufficiency (control) and iron deficiency for 14 days. (**a**) Shoot fresh weight; (**b**) root fresh weight; (**c**) shoot length; (**d**) root length; (**e**) chlorophyll content; and (**f**) lateral root number. Panel (**g**) shows the phenotypes of plants in the treatments. Different lowercase letters above the standard error bars from all graphics indicate significant differences between treatments calculated with two-way ANOVA and Tukey’s test (*p* < 0.5; *n* = 9).

**Figure 3:**
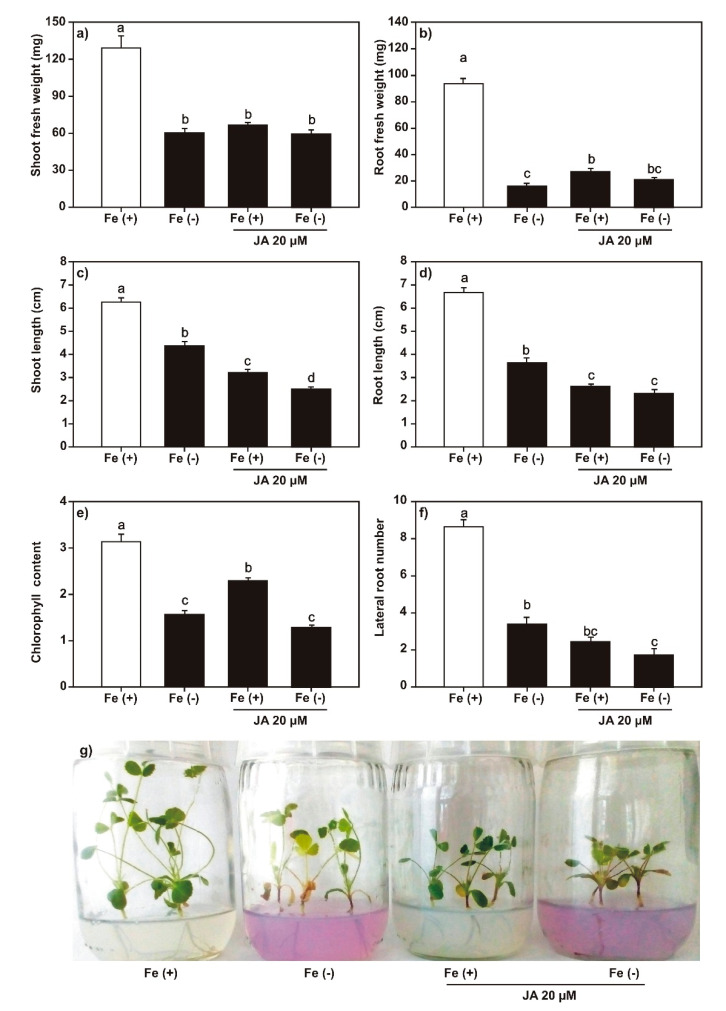
Effect of jasmonic acid (JA) on *Medicago truncatula* growth. The *M. truncatula* plants were cultured in MS medium with JA (20 µM) under both iron sufficiency (control) and iron deficiency or 14 days. (**a**) Shoot fresh weight; (**b**) root fresh weight; (**c**) shoot length; (**d**) root length; (**e**) chlorophyll content; and (**f**) lateral root number. Panel (**g**) shows the phenotypes of plants in the treatments. Different lowercase letters above the standard error bars indicate significant differences between treatments calculated with two-way ANOVA and Tukey’s test (*p* < 0.5; *n* = 9).

We analyzed the effect of SA (100 μM), JA (20 μM), and DMHDA (8 µM) on plant growth under both iron-sufficient and iron-deficient conditions. Plants treated with 100 μM of SA (Figure 2) and 20 μM of JA (Figure 3) showed a decrease in the length and weight of shoots and roots, the number of lateral roots, and chlorophyll content. The effect on growth was greater in plants treated with JA. The combination of SA or JA with iron deficiency had a synergistic effect on the decrease in shoot and root length, number of lateral roots, and chlorophyll content. A less clear effect was observed on the number of trifoliate leaves (Figures S1 and S2).

**Figure 4:**
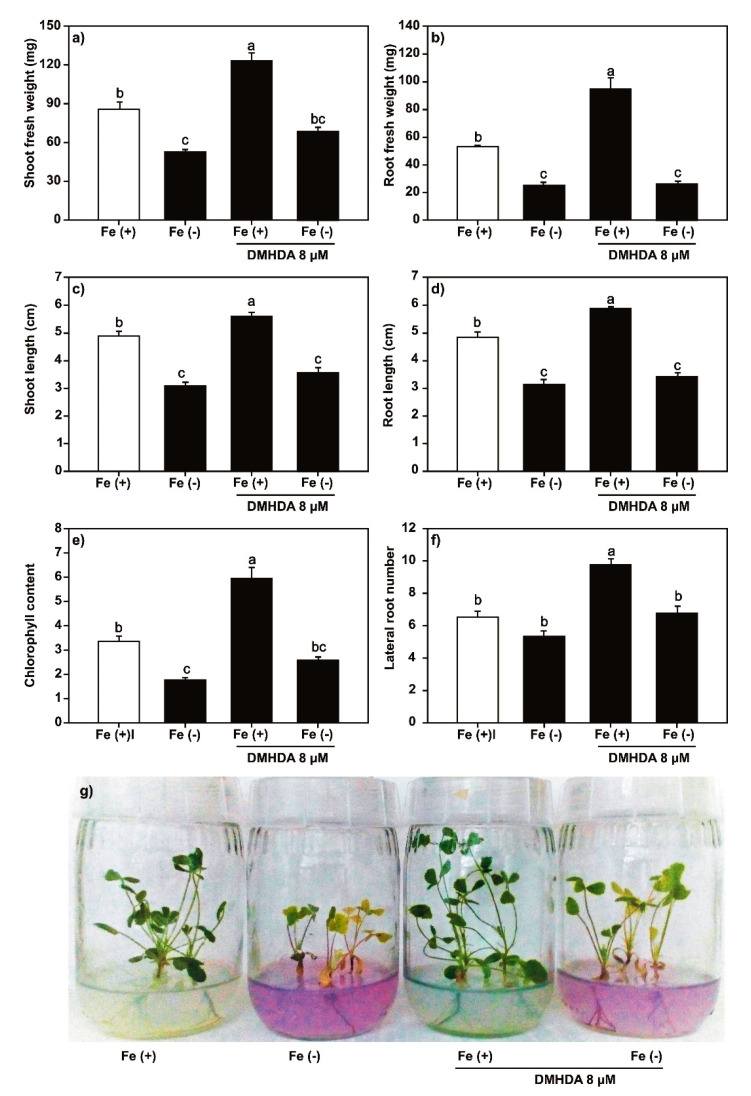
Effect of *N,N*-dimethylhexadecylamine (DMHDA) on *Medicago truncatula* growth. The *M. truncatula* plants were cultured in MS medium with DMHDA (8 µM) under both iron sufficiency (control) and iron deficiency for 14 days. (**a**) Shoot fresh weight; (**b**) root fresh weight; (**c**) shoot length; (**d**) root length; (**e**) chlorophyll content; and (**f**) lateral root number. Panel (**g**) shows the phenotypes of plants in the treatments. Different lowercase letters above the standard error bars indicate significant differences between treatment calculated with two-way ANOVA and Tukey’s test (*p* < 0.5; *n* = 9).

In contrast, the application of DMHDA (8 μM) increased the length and weight of shoots and roots, the number of trifoliate leaves and lateral roots, and chlorophyll content (Figure 4 and Figure S3). Finally, the DMHDA combined with iron deficiency protected the plants against this stress because we observed that the weight and length of shoots, number of lateral roots, and chlorophyll content were significant higher in these plants than plants under iron-deficient conditions without DMHDA.

### 2.3. Effect of Iron Deficiency on the Expression of Iron-Deficiency and Biotic Stress-Response Genes

In the *M. truncatula* genome, we identified the genes *MtbHLH38*, *MtbHLH39*, and *MtFIT* as key genes in iron-deprivation response, *MtNPR4* and *MtWRKY70* as key genes in SAR response, and *MtMYC2* as a key gene in ISR response. Genes were identified by homology with *A. thaliana* genes. All the identified genes showed at least 60% identity with their *A. thaliana* orthologues and the sequences of the characteristic domains of each protein (Table S1). The key genes *MtFRO3* [27,36] (iron-deficiency response), and *MtDef2.1* [37] (ISR response) have been previously described.

**Figure 5:**
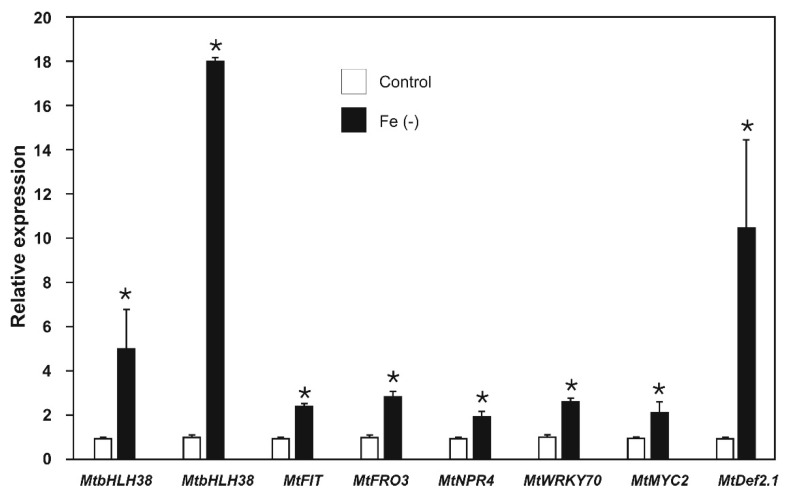
Relative expression of iron uptake and defense genes in *Medicago truncatula* plants grown under both iron-sufficient (control) and iron-deficient conditions for 48 h. Values represent mean ± standard errors of relative expression in reference to controls. Asterisks indicate significant differences between treatments calculated using Student’s t test (*p* < 0.5; *n* = 3).

We proceeded to evaluate the expression of the key genes in the defense pathways and the iron uptake in plants grown under iron deficiency. Previous studies have reported that the expression of iron-deprivation response genes is time dependent [38]; therefore, a time kinetic of *MtFIT* expression was performed (Figure S4). We observed that gene expression peaked at 48 h (although the differences between *MtFIT* expression at different times was not significant); thus, plants were maintained under treatment conditions for 48 h before we measured gene expression. First, we analyzed the expression of key genes under iron-deficient conditions. The expression of *MtbHLH38*, *MtbHLH39*, *MtFIT*, and *MtFRO3* ranged from 2.5- to 18.1-fold higher than that in the respective controls in plants cultured under iron-sufficient conditions (Figure 5). These results showed that the selected genes clearly responded to iron deprivation as expected.

Several previous studies have related iron deficiency to defense responses in plants [23,39,40]. Therefore, the expression of *MtNPR4* and *MtWRKY70* in the SAR pathway, and *MtMYC2* and *MtDef2.1* in the ISR pathway was evaluated in plants subjected to iron-deficient conditions. Genes involved in the SAR response (*MtNPR4* and *MtWRKY70*) showed expressions ranging from 2.2- to 2.7-fold higher than those of the respective controls in plants cultured under iron-sufficient conditions (Figure 5). 

Genes involved in the ISR response (*MtMYC2* and *MtDef2.1*) showed a 2.2- and 10.5-fold increase, respectively (Figure 5). These results indicated that iron deprivation, as well as induction of the expression of genes involved in iron uptake, activates the biotic stress-response genes involved in the signaling pathways of SAR and ISR.

### 2.4. Effect of SA and JA on the Expression of Biotic Stress and Iron-Deficiency Response Genes

**Figure 6:**
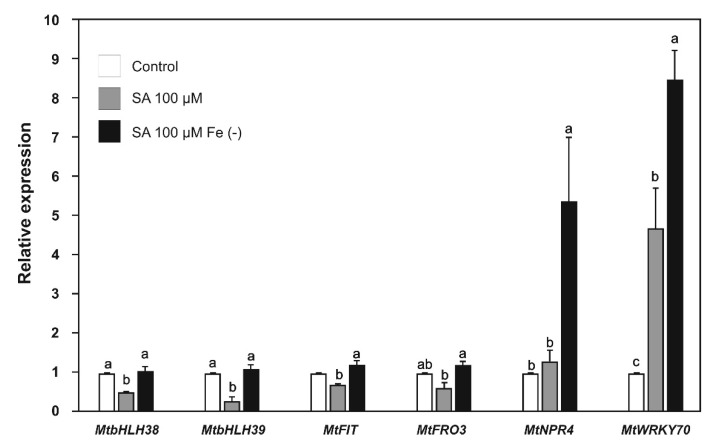
Relative expression of iron uptake and defense genes in *Medicago truncatula* plants grown with salicylic acid (100 µM) under both iron-sufficient and iron-deficient conditions for 48 h. Values represent mean ± standard errors of relative expression in reference to controls. Different lowercase letters indicate significant differences as determined by one-way ANOVA and Tukey’s test (*p* < 0.5; *n* = 3).

**Figure 7:**
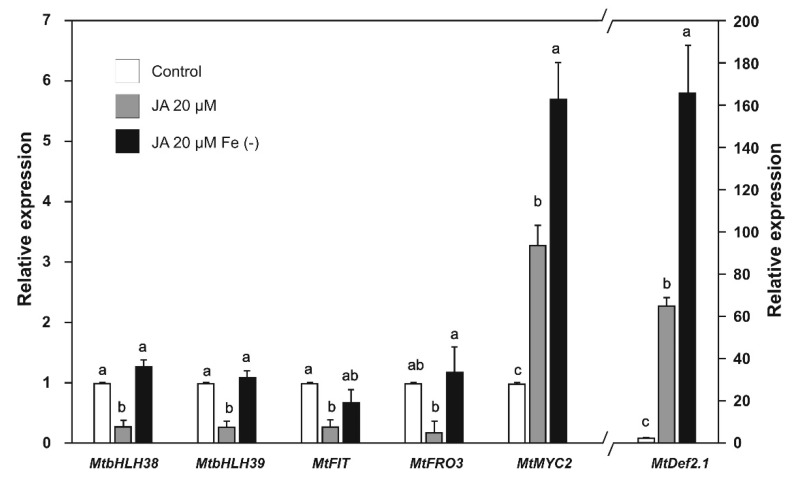
Relative expression of iron uptake and defense genes in *Medicago truncatula* plants grown with jasmonic acid (20 µM) under both iron-sufficient and iron-deficient conditions for 48 h. Values represent mean ± standard errors of relative expression in reference to controls. Values of *MtDef2.1* expression are shown on the axis on the right. Different lowercase letters indicate significant differences as determined by one-way ANOVA and Tukey’s test (*p* < 0.5; *n* = 3).

As we observed that iron deficiency activates defense pathways at the transcriptional level, we decided to evaluate the positive feedback of SA and JA on iron-deficiency response genes. The expression of the *MtbHLH38*, *MtbHLH39*, *MtFIT*, and *MtFRO3* genes was repressed in plants treated with SA (Figure 6) or JA (Figure 7). However, when iron deprivation was combined with SA or JA treatment, the gene repression reverted to similar levels as the control (Figure 6 and Figure 7).

We also analyzed the response of defense pathway genes to their corresponding phytohormone triggers. The *MtNPR4* gene was not induced significantly under iron sufficiency and SA treatment, but the combination of SA and iron deprivation resulted in a 5.4-fold increase in expression (Figure 6). This result suggests an additive effect, which is probably due to the increase in the endogenous levels of this phytohormone under iron deficiency. The expression of the *MtWRKY70* gene showed a 4.7-fold increase in the presence of SA and an 8.5-fold increase under iron deficiency combined with SA (Figure 6).

The expression of the *MtMYC2* gene showed a 3.3-fold increase after application of JA alone, and a 5.7-fold increase after application of JA combined with iron deprivation (Figure 7). The expression of the *MtDef2.1* gene showed a 65.3-fold increase after application of JA alone, and a 166.0-fold increase after application of JA combined with iron deprivation (Figure 7). These results confirm that the genes identified in the present study responded to the phytohormones involved in the defense pathways.

### 2.5. Effect of DMHDA on the Expression of Biotic Stress and Iron-Deficiency Response Genes

**Figure 8:**
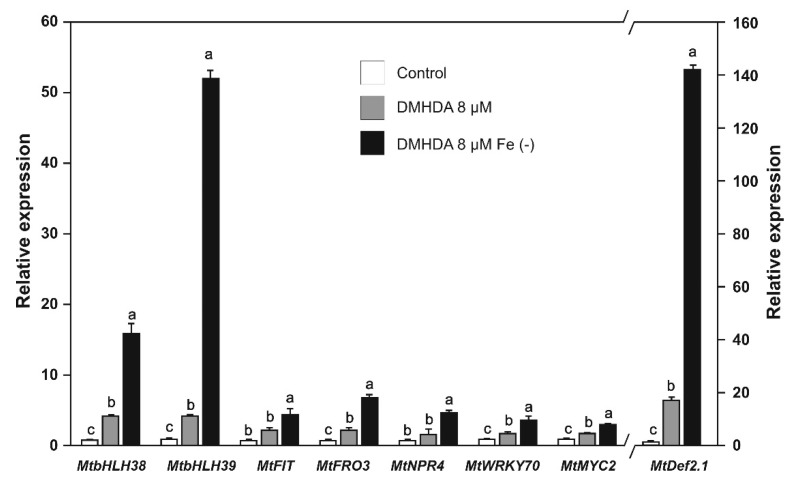
Relative expression of iron uptake and defense genes in *Medicago truncatula* plants grown with DMHDA (8 µM) under both iron-sufficient and iron-deficient conditions for 48 h. Values represent mean ± standard errors of relative expression in reference to controls. Values of *MtDef2.1* expression are referred to secondary axis. Different lowercase letters indicate significant differences as determined by one-way ANOVA and Tukey’s test (*p* < 0.5; *n* = 3).

Aiming to establish a relationship between iron deficiency, SA, JA, and DMHDA, we evaluated the expression of genes involved in iron uptake and biotic stress response in plants treated with DMHDA. The expression of the *MtbHLH38*, *MtbHLH39*, *MtFIT,* and *MtFRO3* genes in DMHDA-treated plants was from 2.4- to 4.4-fold higher than that of the controls, and in plants treated with DMHDA combined with iron deprivation was from 4.7- to 52.2-fold higher than that of the controls (Figure 8). These results showed that DMHDA and iron deprivation have a synergistic effect on iron-deficiency response genes. 

We observed that the plants treated with DMHDA also exhibited transcriptional activation of the biotic stress-response pathways. The *MtNPR4* and *MtWRKY70* genes showed a higher expression compared with the controls (1.9- to 2.1-fold), but plants treated with DMHDA under iron deprivation increased gene expression from 3.8 to 7.5-fold (Figure 8). 

The *MtMYC2* and *MtDef2.1* genes were also induced by DMHDA, but to different magnitudes. The addition of DMHDA under iron deprivation induced a 142-fold increase in the expression of *MtDef2.1*, which was similar to the results from plants treated with JA under iron deprivation (Figure 7 and Figure 8). This result suggests that JA and DMHDA may share a common induction mechanism in ISR responses.

### 2.6. Effect of P. syringae and B. cinerea the Expression of Biotic Stress and Iron-Deficiency Response Genes

**Figure 9:**
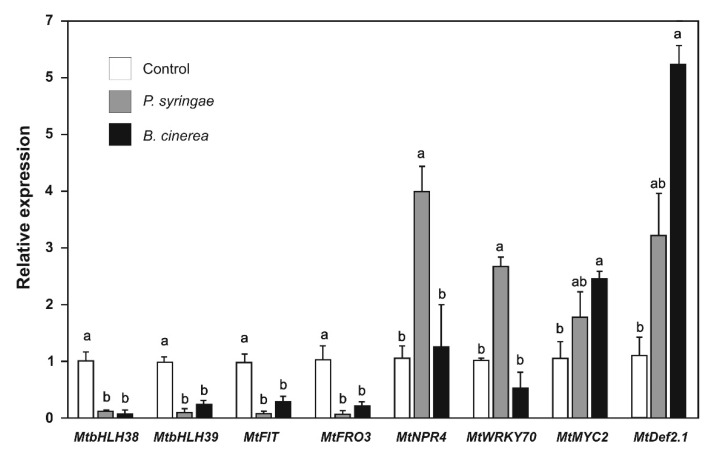
Relative expression of iron uptake and defense genes in *Medicago truncatula* plants inoculated with *Pseudomonas syringae* or *Botrytis cinerea*. Values represent mean ± standard errors of relative expression in reference to controls. Different lowercase letters indicate significant differences as determined by one-way ANOVA and Tukey’s test (*p* < 0.5; *n* = 3).

We evaluated the effect of *P. syringae* and *B. cinerea* inoculation on the expression of biotic stress and iron-deficiency response genes because these pathogens activate the SAR and ISR pathways under natural conditions. The expression of the genes *MtbHLH38*, *MtbHLH39*, *MtFIT*, and *MtFRO3* was strongly repressed when plants were inoculated with *P*. *syringae* (9- to 20-fold) and *B. cinerea* (3.2- to 10-fold) (Figure 9). These results indicate that, as JA and SA did, pathogen inoculation suppressed the iron-deficiency response pathway.

The expression of the SAR and ISR pathway genes was also analyzed. We observed that the expression of the *MtNPR4* and *MtWRKY70* genes were strongly induced after *P. syringae* inoculation, whereas inoculation with *B. cinerea* did not affect the expression of these genes (Figure 9). These results indicate that the genes of the SAR pathway respond to the hemibiotrophic *P. syringae* pathogen. Regarding the genes of the ISR pathway, the expression of *MtMYC2* and *MtDef2.1* was strongly induced after *B. cinerea* inoculation; expression of *MtMYC2* and *MtDef2.1* also increased after *P. syringae* but to a lesser extent (Figure 9).

### 2.7. Effect of DMHDA on Growth of M. truncatula Plants Infected with P. syringae or B. cinerea

**Figure 10:**
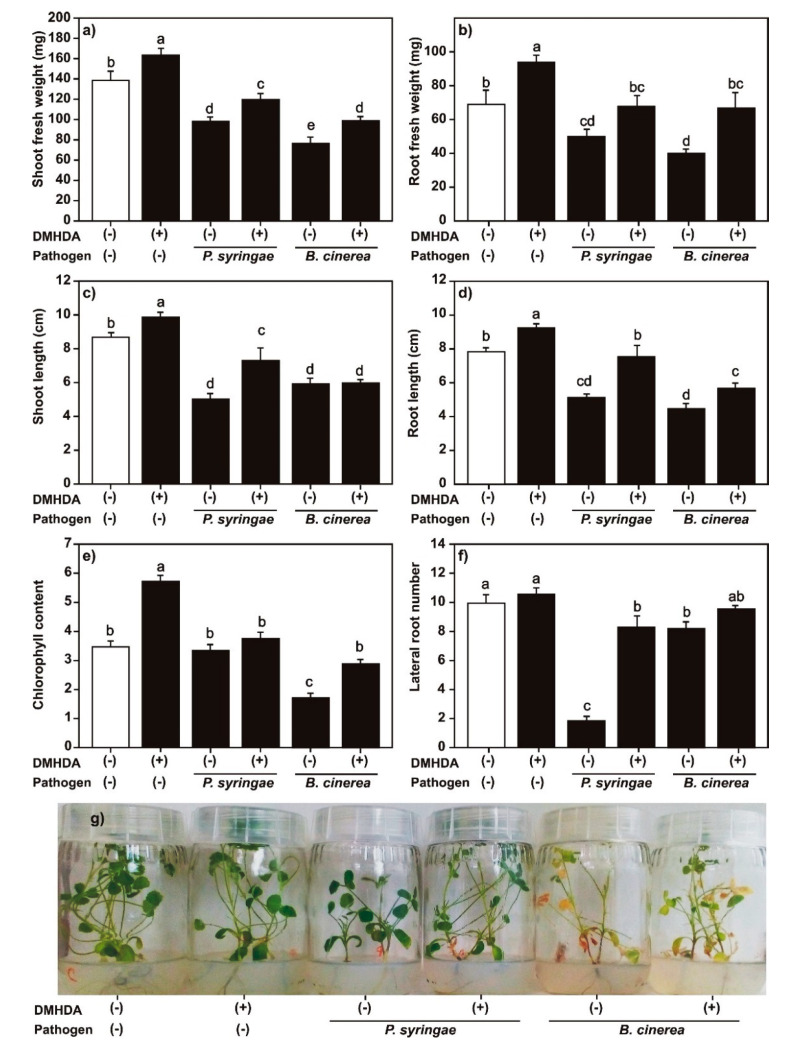
Effect of *N,N*-dimethylhexadecylamine (DMHDA) on growth of plants of *Medicago* infected with *Pseudomonas syringae* or *Botrytis cinerea*. The *M. truncatula* plants were cultured in MS medium with DMHDA (8 µM) and the cultured for 15 days. (**a**) Shoot fresh weight; (**b**) root fresh weight; (**c**) shoot length; (**d**) root length; (**e**) chlorophyll content; and (**f**) lateral root number. Panel (**g**) shows the phenotypes of plants in the treatments. Different lowercase letters above the standard error bars indicate significant differences calculated with two-way ANOVA and Tukey’s test (*p* < 0.5; *n* = 9).

Finally, with the aim to determine if the gene induction produced by DMHDA conferred protection against biotrophic and necrotrophic pathogens, we evaluated the effect of *P. syringae* and *B. cinerea* inoculation on the growth parameters of *M. truncatula* plants cultured with DMHDA. As previously described, uninoculated plants treated with DMHDA (8 μM) increased the length and weight of shoots and roots, and chlorophyll content compared with controls (Figure 10), but there was no significant differences in the number of trifoliate leaves and lateral roots (Figure 10 and Figure S5). Plants inoculated with *P. syringae* or *B. cinerea* had lower shoot and root weights, shoot and root lengths, and number of lateral roots and trifoliate leaves compared with uninoculated controls. Chlorophyll content was strongly affected by *B. cinerea* inoculation but not by *P. syringae* inoculation (Figure 10 and Figure S5). Plants cultured with DMHDA and inoculated with *P. syringae* or *B. cinerea* grew better than plants that were inoculated with the pathogens but cultured without DMHDA, as shown by the growth parameters (shoot and root fresh weight, root length, chlorophyll content, and trifoliate leaf number) (Figure 10 and Figure S5). In particular, plants cultured with DMHDA showed a higher root fresh weight and chlorophyll content than plants cultured without DMHDA (Figure 10b,e). This correlates with a healthier phenotype, comparable with the not infected controls (Figure 10e,g). Plants inoculated with *P. syringae* and cultured with DMHDA also showed a higher shoot length and lateral root number, but plants inoculated with *B. cinerea* with and without DMHDA did not differ in these parameters. These results showed that DMHDA may confer protection to *M. truncatula* plants against pathogens, although this protection was not complete in our experimental system. 

## 3. Discussion

In recent years, response to iron-deficiency stress has been linked to the defensive activation of both SAR and ISR pathways [23]. It has also been shown that the absence of iron decreases the development of symptoms caused by pathogens such as *D. dadantii* [39]. The mechanism underlying this phenomenon remains unknown; however, in *A. thaliana* it is suggested that the subunit of the mediator complex MED16 mediates these responses by interacting with MED25, which interacts with EIN3 and EIL1 (ethylene signaling transcription factors). EIN3 and EIL1 are involved in the JA signaling pathway and act to maintain a normal amount of FIT, which in turn regulates iron homeostasis [41]. Additionally, MED25 interacts with the transcriptional factor MYC2 through the trans-activation domain TAD [42]. MED16 is also involved in the activation of defense pathways through the regulation of SA and JA signaling [43].

In the present study, the expression of genes involved in both iron uptake and defense pathways in the legume model *M. truncatula* plants grown under iron deficiency was evaluated and a relationship was established between iron deficiency and DMHDA. In addition, we compared the effects produced by the phytohormones responsible for activating plant defense (SA and JA) and DMHDA. Before analyzing the expression of the genes, the phenotypes caused by the different conditions were characterized. It was observed that iron deficiency (Figure 1) generated a chlorotic phenotype with lower shoot and root weight and length compared to the control, as well as a decrease in the number of lateral roots. As iron is involved in metabolic reactions in organelles, such as respiration and photosynthesis, as well as in chlorophyll biosynthesis, iron deficiency affects all cellular metabolic processes [44].

In plants treated with SA and JA (Figure 2 and Figure 3), a decrease was observed in the weight and length of shoots and roots and the number of lateral roots and chlorophyll content. This reduction effect was enhanced when phytohormones were combined with iron deprivation. The exogenous application of both SA and JA decreases the length of shoots and roots, and SA affects the photosynthetic capacity of plants [45,46].

Contrary to what was observed with application of SA and JA, the application of DMHDA increased the weight and length of shoots and roots, the number of trifoliate leaves and lateral roots, and chlorophyll content (Figure 4); similar results have been reported in previous studies [26,31]. The combination of DMHDA and iron deficiency resulted in higher values of the analyzed parameters compared with those observed under iron deficiency alone, but in lower values than in the control. DMHDA activates mechanisms involved in iron uptake, such as acidification [31] and the induction of *FRO* genes [32]. Therefore, the protective effect is probably due to this phenomenon.

Currently, there is increasing evidence indicating a sophisticated transcriptional regulatory network that maintain iron homeostasis in plants. In *A. thaliana*, 16 of the transcriptional factors integrating this network belong to the bHLH family. The bHLH transcriptional factors acts as homo- or heterodimers to regulate the expression of their target genes that frequently are other *bHLH* genes [47]. The bHLH transcription factors are also involved in the JA signaling pathway. In *A. thaliana* plants MYC2 (bHLH6) is often considered as a central transcriptional factor of the JA signaling pathway [42] and acts as a JA-dependent repressor of FIT [47]. The SA and JA signaling pathways pathway also cross-talk, and it has been pointed out that the transcriptional factor WRKY70 is involved in this interaction since it represses the JA-responsive genes, and activates the SA-responsive genes [48].

In the present study, we identified in the *M. truncatula* genome the orthologues of the key transcription factor genes *bHLH38*, *bHLH39*, and *FIT* (iron-deprivation response) [35,49]; *WRKY70* (SAR response) [1,48]; and *MYC2* (ISR response) [1,42], and employed them to analyze their signaling pathways together with *MtFRO3* [27], *MtNPR4* (this work), and *MtDef2.1* [37] placed downstream on those respective signaling cascades. We observed that iron deprivation up-regulated the expression of *MtbHLH38*, *MtbHLH39*, *MtFIT,* and *MtFRO3* genes (Figure 5), and had a greater effect on *MtbHLH38* and *MtbHLH39* with 5-fold and 18-fold increases, respectively. In previous reports, higher induction results were observed in the orthologues of these genes over others involved in iron deficiency [35], which indicates that these genes play a key role in activating iron uptake response.

The genes of SAR and ISR were also induced by iron deprivation (Figure 5). We observed induced expression for all defense genes, which indicates that iron deficiency regulates this process at the transcriptional level. Iron deficiency increases the endogenous levels of JA [50] and SA [51]; thus, it can be speculated that the increased expression of defense genes is due to induced synthesis of JA and SA triggered by iron deficiency. Additionally, the expression of MYB72 a transcriptional factor essential to mounting ISR responses against phytopathogen microorganisms [52] is driven by FIT1 in *A. thaliana* [53].

The application of SA and JA prevented the up-regulation of the *MtbHLH38*, *MtbHLH39*, *MtFIT,* and *MtFRO3* genes when the plants were grown under iron deficiency combined with these phytohormones. In *A. thaliana,* SA decreased the expression of *FRO2* [54], whereas JA decreased *FIT (bHLH29), bHLH38, bHLH39*, *FRO2,* and *IRT* gene expression [55,56]. However, other studies have reported that SA increased the expression of the *bHLH38* and *bHLH39* genes [57], while in *Oryza sativa*, JA is involved in the positive regulation of the *IDEF1* and *IBP1.1* genes, which are involved in the activation of the iron uptake response [50]. Our results from the *M. truncatula* model support the negative regulation of iron uptake by SA and JA.

The effect of SA and JA on defense genes was analyzed. The *MtMYC2* and *MtDef2.1* genes were clearly induced by JA, and this induction was potentiated by iron deprivation as expected by ISR genes [23,53]. In the same way, SA induced *MtWRKY70* gene expression and iron deprivation enhanced this induction as expected [39,58]; however, *MtNPR4* was significantly induced when SA was combined with iron deprivation, and this was probably due to the increase in endogenous levels of SA triggered by iron deficiency. Previous research has shown that the expression of some *PR*s genes of different plants is activated by a combination of phytohormones, such as SA/methyl ester JA (MeJA) [59] or MeJA alone [60]. 

DMHDA induced *MtbHLH38*, *MtbHLH39*, *MtFIT,* and *MtFRO3* gene expression (Figure 8), and this induction was potentiated when DMHDA was combined with iron deprivation. Similar behavior was previously observed in the *M. truncatula* system in experiments on rhizosphere acidification (iron-deficiency response) [31]. Thus, our results showed that DMHDA also regulates the response to iron deficiency at the transcriptional level as expected. Additionally, DMHDA activates the two defense pathways, since the plants treated with this compound showed a marginal increase in *MtNPR4*, *MtWRKY70*, and *MtMYC2*, gene expression but a clear increase in *MtDef2.1.* The gene expression of all these genes was potentiated with the combination of iron deprivation and DMHDA. In particular, *MtDef2.1* showed considerable induction of expression after DMHDA treatment under iron deprivation, which was comparable to that of JA application under iron deprivation, suggesting that DMHDA may act through the JA pathway.

To produce not an exogenous but a physiological induction of SAR and ISR pathways, we infected the plants with *P. syringae* or *B. cinerea* and evaluated the iron-deprivation response, SAR pathway, and ISR pathway gene expression, and we obtained similar results to SA and JA addition to plants (Figure 9). The presence of these pathogens had a negative effect on the expression of genes involved in iron uptake, but *B. cinerea* triggered ISR pathway gene expression, whereas *P. syringae* induced SAR gene expression. These results demonstrated that, under experimental conditions that emulate natural conditions, the gene expression pattern produced by the addition of SA and JA was conserved. Furthermore, *M. truncatula* plants treated with DMHDA and infected with *P. syringae* and *B. cinerea* were healthier and grew better than plants not treated with DMHDA but infected with pathogens. This supports the idea that under real infection, defense pathways induced by DMHDA conferred protection against biotrophic and necrotrophic pathogens.

**Figure 11:**
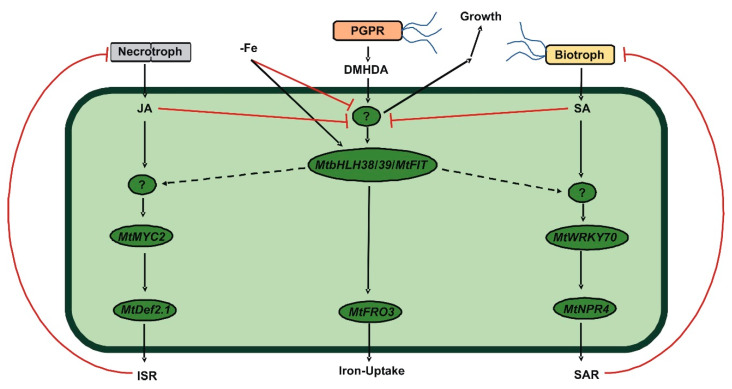
Proposed model of cross-talk between systemic acquired resistance (SAR), induced systemic resistance (ISR), and iron-deprivation response pathways mediated by *N,N*-dimethylhexadecylamine (DMHDA) in *Medicago truncatula*. Necrotrophic pathogens trigger jasmonic acid (JA) synthesis in plant cells, which downstream induces the expression of *MtMYC2* and *MtDef2.1* as ISR responses, which in turn inhibit the necrotrophic pathogen attack. Biotrophic pathogens trigger salicylic acid (SA) synthesis in plant cells, which downstream induces the expression of *MtWRKY70* and *MtPR4* as SAR responses, which in turn inhibit the biotrophic pathogen attack. Some plant growth promoting rhizobacteria (PGPR) produce DMHDA that activates a regulatory element upstream of *MtbHLH38*/*39*/*MtFIT* that induces *MtFRO3* as an iron-deprivation response. This regulatory element also promotes plant growth. Iron deprivation induces *MtbHlH38*/*39*/*MtFIT* but inhibits plant growth, probably through a regulatory element located upstream of *MtbHlH38*/*39*/*MtFIT*. JA and SA inhibit plant growth and iron-deprivation responses upstream of *MtbHlH38*/*39*/*MtFIT. MtbHlH38*/*39*/*MtFIT* induces ISR upstream of *MtMYC2* and SAR upstream of *MtPR4.*

It is proposed that plants maintain an energetic balance between growth and defense [61]. In this way, the stimulation of defense pathways has a detrimental effect on growth [62,63]. Other bacterial VOCs, such as 2,3-butanediol, have the ability to activate both SAR and ISR defense pathways simultaneously with plant growth promotion [64], as DMHDA did. DMHDA increases the expression of *AtLOX2*, a gene that responds to JA and is involved in JA biosynthesis [33], and both DMHDA and JA are antagonized by kinetin and correlated with an inhibition of cytokinin-related *ARR5*::*GUS* and *TCS*::*GFP* expression in *A. thaliana* [65]. Thus, we hypothesized that the effects of DMHDA in plants trigger the JA pathway. However, the results of the present study revealed a different process. The addition of SA or JA inhibited the growth of all the parameters that we recorded, and growth was more strongly inhibited when the addition of SA or JA was under iron-deficient conditions. However, in plants treated with DMHDA, growth was promoted, and when iron deprivation or infection with pathogens were combined with DMHDA treatment, the iron or biotic stresses effects were mitigated. Iron deprivation and DMHDA induced SAR, ISR, and iron-deficiency gene expression, especially when combined; however, SA and JA inhibited iron response genes (Figure 11). 

In conclusion, we identified key genes in the defense and iron-deprivation responses of the legume model *M. truncatula* and reaffirmed the interactions between them. Taken together, our results showed that the VOC DMHDA produced by PGPR activates defense and iron-deprivation pathways, and exhibits a growth promoting effect unlike conventional phytohormones, highlighting that DMHDA does not mimic JA but induces an alternative pathway, revealing a novel aspect in the complex interactions between biotic and abiotic stresses.

## 4. Materials and Methods 

### 4.1. M. truncatula Seed Germination

The *M. truncatula* ecotype Jemalong A17 was used in the present study. Seeds were submerged in a tube with 1 mL of sulfuric acid and were constantly shaken for 8 min. The excess of acid was removed, and the seeds were rinsed seven times with sterile deionized water. Sterilization was carried out with a 12% sodium hypochlorite solution for 2 min. Subsequently, the seeds were rinsed five times with sterile deionized water. The seeds were transferred to Petri dishes with Murashige and Skoog (MS) 0.2× medium at 6.5 pH with 6 g of agar L^−1^ and vernalized for 1 day at 4 °C. Finally, 2 days after germination, the seedlings were transplanted into glass jars with 30 mL of MS to apply the corresponding treatments.

### 4.2. MS Medium Preparation 

The MS medium used for germination and growth was prepared as follows. In 1 L of water, we added 200 µl of solution 1 (25 g L^−1^ of CaCl_2_), 2 mL of solution 2 (9.25 g L^−1^ of MgSO_2_ and 4.25 g L^−1^ of KH_2_PO_4_), 1 mL of solution 3 (0.5 g L^−1^ of FeSO_4_ and 0.7 g L^−1^ of NaEDTA), 200 μL of solution 4 (1.69 g L^−1^ of MnSO_4_, 1.05 g L^−1^ of ZnSO_4_, 0.62 g L^−1^ of H_3_BO_3_, 0.83 g L^−1^ of KI, 0.025 g L^−1^ of Na_2_MoO_4_, 0.0025 g L^−1^ of CuSO_4_, and 0.0025 g L^−1^ CoCl_2_), 2 mL of solution 5 (0.5 g L^−1^ of glycine, 0.0125 g L^−1^ of pyridoxine, 0.025 g L^−1^ of nicotinic acid, 0.0025 g L^−1^ of thiamine, and 1.5 g L^−1^ of myo-inositol), and 2 mL of solution 6 (41.25 g L^−1^ of NH_2_NO_3_ and 47.5 g L^−1^ KNO_3_). To induce iron deficiency, solution 3 was omitted, and ferrozine was added to have a final concentration of 100 µM. Finally, the pH of the medium was adjusted to 6.5 and sterilized for 20 min in a pressure vessel. 

### 4.3. Chemicals

SA, JA, and DMHDA were purchased from Sigma-Aldrich. SA and JA and were dissolved in water, and DMHDA in ethanol. Equal volumes of solvent used in treatments were added to controls.

### 4.4. Growth and Gene Expression Evaluation

Two days after germination, the seedlings were transplanted into glass jars containing 30 mL of MS. Three flasks were used per treatment, with three plants in each. Control (+Fe) and iron-deficiency (−Fe) conditions were used. Additionally, SA, JA, or DMHDA were added to the MS medium to a final concentration of 100 μM [57], 20 μM, and 8 μM [26], respectively, under both iron-sufficient and iron-deficient conditions. To evaluate the growth parameters, the plants were kept in an AR-66L2 growth chamber (Percival Scientific, Inc. Perry, IA, USA) for 14 days with a photoperiod of 16-h light/8-h dark and a light intensity of 200 µmol m^−2^ s^−1^ at 22 °C. After 14 days, the roots and shoots were measured and weighed, and then lateral roots, leaves, and chlorophyll content were quantified. To analyze gene expression, the germinated plants were grown for three days in MS and then transferred for 48 h to their respective treatment or to MS without phytohomones (controls). After 48 h, total RNA was extracted from the whole plant.

### 4.5. P. syringae and B. cinerea Inoculation

*M. truncatula* plants were inoculated according to a previously described protocol [30]. Plants were inoculated with 10 μM of 1 × 10^5^ spores of *B. cinerea* and 10 μM of 1 × 10^7^ CFU of *P. syringae* 5 days after being transferred to MS. Two days post inoculation, gene expression was evaluated. For *M. truncatula* growth assays, plants were transferred to MS without (controls) or with DMHDA and cultured for 5 days. Then plants were inoculated with *B. cinerea* or *P. syringae* and cultured for 15 days. 

### 4.6. Growth Analysis and Quantification of Chlorophyll Content in M. truncatula

Roots and aerial parts were weighed using an analytical balance, and then their length was measured. The lateral roots and leaves were quantified manually. Chlorophyll content was quantified as previously described [32] using a CCM-200 chlorophyll meter (Opti-Sciences, Inc., Hudson, NH, USA) based on the rate of transmitted radiation (940 and 660 nm) through a leaf in arbitrary units.

### 4.7. Identification of Defense and Iron-Deficiency Genes

The genes evaluated in the present study were identified in the genome of *M. truncatula* (http://blast.jcvi.org/er-blast/index.cgi?project=mtbe) by performing a BLAST search against genes previously reported in *A. thaliana*. Additionally, the domains of the selected sequences were identified using the NCBI tool “Conserved domain search” (https://www.ncbi.nlm.nih.gov/Structure/cdd/wrpsb.cgi) (Table S1).

### 4.8. RNA Extraction and cDNA Synthesis

The whole plant was macerated with liquid nitrogen. Total RNA was extracted with the TRI reagent (Catalog T9424, Sigma-Aldrich). Prior to its use, the RNA was treated with DNase to remove DNA residues. The samples were run on a 1% agarose gel at 90 V to determine the integrity of the molecule and absorbance. The quality and quantity of RNA was assessed using a NanoDrop 1000 spectrophotometer (Thermo Scientific, Rockford, IL, USA) to calculate the ratio of absorbance at 260 nm to absorbance at 280 nm (Table S2) and by examination on agarose gel electrophoresis (1% agarose gel at 90 V) (Figure S6). Finally, cDNA was synthesized according to the specifications of the “SuperScript™ First-Strand Synthesis System for RT-PCR” kit (Life Technologies/Gibco-BRL., Carlsbad, CA, USA).

### 4.9. RT-qPCR

RT-qPCR was performed in triplicate for each treatment and gene using the ABI StepOne™ System thermocycler (Applied Biosystems, Foster City, CA, USA). The oligonucleotides for the genes were designed using the NCBI tool “First Designing Tool” (https://www.ncbi.nlm.nih.gov/tools/primer-blast) and are listed in Table S3. The RT-qPCR analysis was carried out with SYBR-Green PCR Master Mix (Applied Biosystems) in a volume containing 5 μL SYBR-Green PCR Master Mix, 1 μL of the oligonucleotide mixture (forward and reverse), 2 μL of cDNA, and 3 μL of deionized sterile water. The thermal cycling protocol was as follows: 95 °C for 10 min, 40 cycles at 95 °C for 15 s, and 60 °C for 30 s. To verify the amplification of single, specific target cDNA, a dissociation curve analysis was included according to the thermal profile, as suggested by the manufacturer (Applied Biosystems). To prepare the dissociation curve, the reaction was terminated at 95 °C for 15 s followed immediately by annealing and extension at 60 °C for 1 min; finally, the temperature was increased to 95 °C at a rate of 0.3 °C s^−1^. A specific target cDNA corresponding to a single dissociative peak was obtained in all the cases (Figure S7).

The amount of RNA in each sample was normalized using actin as the reference gene. Finally, gene expression was evaluated using the comparative 2^−ΔΔCt^ method [66].

### 4.10. Statistical Analysis

The results were analyzed using Student’s t test or with one-way or two-way analysis of variance (ANOVA) and Tukey’s test for multiple comparisons (*p* < 0.05). Growth parameter experiments were performed with nine biological replicates. Gene expression was analyzed with three composite biological samples. Each composite sampled consisted of three plants. Each experiment was carried out at least twice with similar results.

## Supplementary Materials

The following are available online at https://www.mdpi.com/2223-7747/9/5/624/s1, Figure S1: Effect of salicylic acid (SA) on *Medicago truncatula* trifoliate leaf number, Figure S2: Effect of jasmonic acid (JA) on *Medicago truncatula* trifoliate leaf number, Figure S3: Effect of *N*,*N*-dimethylhexadecylamine (DMHDA) on *Medicago truncatula* trifoliate leaf number, Figure S4: Time kinetics of MtFIT gene expression, Figure S5: Effect of *N*,*N*-dimethylhexadecylamine (DMHDA) and *Pseudomonas syringae* or *Botrytis cinerea* infection on *Medicago truncatula* trifoliate leaf number, Figure S6: Representative images of RNA samples run on 1% agarose gel, Figure S7: Dissociation curves produced by RT-qPCR amplicons of genes listed in Table S1, Table S1: Genes identified in the present study, Table S2: Ratio of absorbance at 260 nm to absorbance at 280 nm of RNA samples used in the RT-qPCR measurements, Table S3: List of oligonucleotides employed in RT-qPCR.

## References

[1] Pieterse C.M.J., Zamioudis C., Berendense R.L., Weller D.M., Van Wees S.C.M., Baker P.A.H.M. (2014). Induced systemic resistance by beneficial microbes. Annu. Rev. Phytopathol..

[2] Boller T., Felix G. (2009). A renaissance of elicitors: Perception of microbe-associated molecular patterns and danger signals by pattern-recognition receptors. Annu. Rev. Plant. Biol..

[3] Acevedo F.E., Rivera-Vega L.J., Chung S.H., Ray S., Felton G.W. (2015). Cues from chewing insects the intersection of DAMPs, HAMPs, MAMPs and effectors. Curr. Opin. Plant Biol..

[4] Choi H.W., Klessig D.F. (2016). DAMPs, MAMPs, and NAMPs in plant innate immunity. BMC Plant Biol..

[5] Jones J.D.G., Dangl J.L. (2006). The plant immune system. Nature.

[6] Shah J., Zeier J. (2013). Long-distance communication and signal amplification in systemic acquired resistance. Front. Plant Sci..

[7] Pieterse C.M.J., Leon-Reyes A., Van der Ent S., Van Wees S.C.M. (2009). Networking by small-molecule hormones in plant immunity. Nat. Chem. Biol..

[8] De Vleesschauwer D.H., Ofte M. (2009). Rhizobacteria-induced systemic resistance. Adv. Bot. Res..

[9] Van Loon L.C., Rep M., Pieterse C.M.J. (2010). Significance of inducible defense-related proteins in infected plants. Annu. Rev. Phytopathol..

[10] Bürger M., Chory J. (2019). Stressed out about hormones: How plants orchestrate immunity. Cell Host Microbe.

[11] Glazebrook J. (2005). Contrasting mechanisms of defense against biotrophic and necrotrophic pathogens. Annu. Rev. Phytopathol..

[12] Mimmo T., Del Buono D., Terzano R., Tomasi N., Vigani G., Crecchio R., Pinton R., Zocchi G., Cesco S. (2014). Rhizospheric organic compounds in the soil-microorganism-plant system: Their role in iron availability. Eur. J. Soil Sci..

[13] Guerinot M.L., Yi Y. (1994). Iron: Nutritious, noxious, and not readily available. Plant Physiol..

[14] Abadía J., Vázquez S., Rellán-Álvarez R., El-Jendoubi H., Abadía A., Álvarez-Fernández A., López-Millán A.F. (2011). Towards a knowledge-based correction of iron chlorosis. Plant Physiol. Biochem..

[15] Santi S., Schmidt W. (2009). Dissecting iron deficiency-induced proton extrusion in Arabidopsis roots. New Phytol..

[16] Robinson N.J., Procter C.M., Connolly E.L., Guerinot M.L. (1999). A ferric-chelate reductase for iron uptake from soils. Nature.

[17] Eide D., Broderius M., Fett J., Guerinot M.L. (1996). A novel ironregulated metal transporter from plants identified by functional expression in yeast. Proc. Natl. Acad. Sci. USA.

[18] Colangelo E.P., Guerinot M.L. (2004). The essential bHLH protein FIT1 is required for the iron deficiency response. Plant Cell.

[19] Yuan Y., Wu H., Wang N., Li J., Zhao W., Du J., Wang D., Ling H.Q. (2008). FIT interacts with AtbHLH38 and AtbHLH39 in regulating iron uptake gene expression for iron homeostasis in *Arabidopsis*. Cell Res..

[20] Wang N., Cui Y., Liu Y., Fan H., Du J., Huang Z., Yuan Y., Wu H., Ling H. (2013). Requirement and functional redundancy of Ib subgroup bHLH proteins for iron deficiency responses and uptake in *Arabidopsis thaliana*. Mol. Plant..

[21] Rodríguez-Celma J., Lin W.D., Fu G.M., Abadía J., López-Millán A.F., Schmidt W. (2013). Mutually exclusive alterations in secondary metabolism are critical for the uptake of insoluble iron compounds by Arabidopsis and *Medicago truncatula*. Plant Physiol..

[22] Joshi J.R., Burdman S., Lipsky A., Yedidia I. (2015). Effects of plant antimicrobial phenolic compounds on virulence of the genus *Pectobacterium*. Res. Microbiol..

[23] Koen E., Trapet P., Brulé D., Kulik A., Klinguer A., Atauri-Miranda L., Meunier-Priest R., Boni G., Glauser G., Mauch-Mani B. (2014). β-Aminobutyric acid (BABA)-induced resistance in *Arabidopsis thaliana*: Link with iron homeostasis. Mol. Plant Microbe Interact..

[24] Segond D., Dellagi A., Lanquar V., Rigault M., Patrit O., Thomine S., Expert D. (2009). NRAMP genes function in *Arabidopsis thaliana* resistance to Erwinia chrysanthemi infection. Plant J..

[25] Zhou C., Guo J., Zhu L., Xiao X., Xie Y., Zhu J., Ma Z., Wang J. (2016). *Paenibacillus polymyxa* BFKC01 enhances plant iron absorption via improved root systems and activated iron acquisition mechanisms. Plant Physiol. Biochem..

[26] Velázquez-Becerra C., Macías-Rodríguez L.I., López-Bucio J., Altamirano-Hernández J., Flores-Cortez I., Valencia-Cantero E. (2011). A volatile organic compound analysis from *Arthrobacter agilis* identifies dimethylhexadecylamine, an amino-containing lipid modulating bacterial growth and *Medicago sativa* morphogenesis in vitro. Plant Soil.

[27] Montejano-Ramírez V., Martínez-Camara R., García-Pineda E., Valencia-Cantero E. (2018). Rhizobacterium *Arthrobacter agilis* UMCV2 increases organ-specific expression of *FRO* genes in conjunction with genes associated with the systemic resistance pathways of *Medicago truncatula*. Acta Physiol. Plant..

[28] Martínez-Medina A., VanWees S.C.M., Pieterse C.M.J. (2017). Airborne signals from *Trichoderma* fungi stimulate iron uptake responses in roots resulting in priming of jasmonic acid-dependent defences in shoots of *Arabidopsis thaliana* and *Solanum lycopersicum*. Plant Cell Environ..

[29] Orozco-Mosqueda M.C., Macías-Rodríguez L.I., Santoyo G., Flores-Cortez I., Farías-Rodríguez R., Valencia-Cantero E. (2013). *Medicago truncatula* increases its iron-uptake mechanisms in response to volatile organic compounds produced by *Sinorhizobium meliloti*. Folia Microbiol..

[30] Hernández-León R., Rojas-Solís D., Contreras-Pérez M., Orozco-Mosqueda M.C., Macías-Rodríguez L.I., Reyes-de la Cruz H., Valencia-Cantero E., Santoyo G. (2015). Characterization of the antifungal and plant-growth promoting effects of diffusible and volatile organic compoundd produced by *Pseudomonas fluorescens* strains. Biol. Control.

[31] Orozco-Mosqueda M.C., Velázquez-Becerra C., Macías-Rodríquez L.I., Santoyo G., Flores-Corez I., Alfaro-Cuevas R., Valencia-Cantero E. (2013). *Arthrobacter agilis* UMCV2 induces iron acquisition in *Medicago truncatula* (strategy I plant) in vitro via dimethylhexadecylamine emission. Plant Soil.

[32] Castulo-Rubio D.Y., Alejandre-Ramírez N., Orozco-Mosqueda M.C., Santoyo G., Macias-Rodríguez L.I., Valencia-cantero E. (2015). Volatile organic compounds produced by the rhizobacterium *Arthrobacter agilis* UMCV2 modulate *Sorghum bicolor* (strategy II plant) morphogenesis and *SbFRO1* transcription in vitro. J. Plant Growth Regul..

[33] Raya-González J., Velázquez-Becerra C., Barrera-Ortíz S., López-Bucio J., Valencia-Cantero E. (2017). *N*,*N*-dimethyl hexadecylamine and related amines regulate root morphogenesis via jasmonic acid signaling in *Arabidopsis thaliana*. Protoplasma.

[34] Hernández-Calderón E., Aviles-Garcia M.A., Castulo-Rubio D.Y., Macías-Rodríguez L., Montejano-Ramírez V., Santoyo G., López-Bucio J., Valencia-Cantero E. (2018). Volatile compounds from beneficial or pathogenic bacteria differentially regulate root exudation, transcription of iron transporters, and defense signaling pathways in *Sorghum bicolor*. Plant Mol. Biol..

[35] Zhang J., Liu B., Mengshu L., Feng D., Jin H., Wang P., Liu J., Xiong F., Wang J., Wang H.B. (2015). The bHLH transcription factor bHLH104 interacts with IAA-LEUCINE RESISTANT3 and modulates iron homeostasis in Arabidopsis. Plant Cell.

[36] Orozco-Mosqueda M.C., Santoyo G., Farías-Rodríguez R., Macías-Rodríguez L.I., Valencia-Cantero E. (2012). Identification and expression analysis of multiple *FRO* gene copies in *Medicago truncatula*. Genet. Mol. Res..

[37] Hanks J.N., Snyder A.K., Graham M.A., Shah R.K., Blaylock L.A., Harrison M.J., Shah D.M. (2005). Defensin gene family in *Medicago truncatula*: Structure, expression and induction by signal molecules. Plant Mol. Biol..

[38] Andaluz S., Rodríguez-Chelma J., Abadía A., Abadía J., López-Milán A.F. (2009). Time course induction of several key enzymes in *Medicago truncatula* roots in response to Fe deficiency. Plant Physiol. Biochem..

[39] Kieu N.P., Aznar A., Segond D., Rigault M., Simond-Côte E., Kunz C., Soulie M.C., Expert D., Dellagi A. (2012). Iron deficiency affects plant defence responses and confers resistance to *Dickeya dadantii* and *Botrytis cinereal*. Mol. Plant Pathol..

[40] Hsiao P., Cheng C., Koh K.W., Chan M. (2017). The *Arabidopsis* defensin gene, *AtPDF1*.1, mediates defence against Pectobacterium carotovorum subsp. carotovorum via an iron-withholding defence system. Sci. Rep..

[41] Yang Y., Ou B., Zhang J., Si W.J., Gu H., Qin G., Qu L. (2014). The Arabidopsis Mediator subunit MED16 regulates iron homeostasis by associating with EIN3/EIL1 through subunit MED25. Plant J..

[42] Goossens J., Mertens J., Goossens A. (2017). Role and functioning of bHLH transcription factors in jasmonate signaling. J. Exp. Bot..

[43] Zhang X., Wang C., Zhang Y., Sun Y., Mou Z. (2012). The *Arabidopsis* mediator complex subunit 16 positively regulates salicylate-mediated systemic acquired resistance and jasmonate/ethylene-induced defense pathways. Plant Cell.

[44] Vigani G., Zocchi G., Bashir K., Philippar K., Briat J.F. (2013). Signal from chloroplasts and mitochondria for iron homeostasis regulation. Trends Plant Sci..

[45] Pancheva T.V., Popoya L.P., Uzunoya A.N. (1996). Effects of salicylic acid on growth and photosynthesis in barley plants. J. Plant Physiol..

[46] Jang G., Chang S.H., Um T.Y., Lee S., Kim J.K., Choi Y.D. (2017). Antagonistic interaction between jasmonic acid and cytokinin in xylem development. Sci. Rep..

[47] Gao F., Robe K., Gaymard F., Izquierdo E., Dubos C. (2019). The transcriptional control of iron homeostasis in plants: A tale of bHLH transcription factors?. Front. Plant Sci..

[48] Li J., Brader G., Kariola T., Palva E.T. (2006). WRKY70 modulates the selection of signaling pathways in plant defense. Plant J..

[49] Li X., Zhang H., Ai Q., Liang G., Yu D. (2016). Two bHLH transcription factors, bHLH34 and bHLH104, regulate iron homeostasis in *Arabidopsis thaliana*. Plant Physiol..

[50] Kobayashi T., Itai R.N., Senoura T., Oikawa T., Ishimaru Y., Ueda M., Nakanishi H., Nishizaw N.K. (2016). Jasmonate signaling is activated in the very early stages of iron deficiency responses in rice roots. Plant Mol. Biol..

[51] Shen C., Yang Y., Liu K., Zhang L., Guo H., Sun T., Wang H. (2016). Involvement of endogenous salicylic acid in iron-deficiency responses in Arabidopsis. J. Exp. Bot..

[52] Van der Ent S., Verhagen B.W.M., Van Doorn R., Bakker D., Verlaan M.G., Pel M.J.C., Joosten R.G., Proveniers M.C.G., Van Loon L.C., Ton J. (2008). MYB72 is required in early signaling steps of rhizobacteria-induced systemic resistance in Arabidopsis. Plant Physiol..

[53] Romera F.J., García M.J., Lucena C., Martínez-Medina A., Aparicio M.A., Ramos J., Alcántara E., Angulo M., Pérez-Vicente R. (2019). Induced systemic resistance (ISR) and Fe deficiency responses in dicot plants. Front. Plant Sci..

[54] Maurer F., Naranjo Arcos M.A., Bauer P. (2014). Responses of a triple mutant defective in three iron deficiency-induced BASIC HELIX-LXOOP-HELIX genes of the subgroup Ib(2) to iron deficiency and salicylic acid. PLoS ONE.

[55] Maurer F., Mueller S., Bauer P. (2011). Suppression of Fe deficiency gene expression by jasmonate. Plant Physiol. Biochem..

[56] Cui Y., Chen C.-L., Cui M., Zhou W.-J., Wu H.-L., Ling H.-Q. (2018). Four IVa bHLH transcription factors are novel interactors of FIT and mediate JA inhibition of iron uptake in *Arabidopsis*. Mol. Plant.

[57] Kang H.G., Foley R.C., Oñate-Sánchez L., Lin C., Singh K.B. (2003). Target genes for OBP3, a Dof transcription factor, include novel basic helix-loop-helix domain proteins inducible by salicylic acid. Plant J..

[58] Abeysinghe J.K., Lam K.-M., Ng D.W.-K. (2019). Differential regulation and interaction of homoeologous WRKY18 and WRKY40 in Arabidopsis allotetraploids and biotic stress responses. Plant J..

[59] Xu Y., Chang P., Liu D., Narasimhan M.L., Raghothama K.G., Hasegawa P.M., Bressan R.A. (1994). Plant defense genes are synergistically induced by ethylene and methyl jasmonate. Plant Cell.

[60] Mei C., Qi M., Sheng G.D., Yang Y. (2006). Inducible overexpression of a rice allene oxide synthase gene increases the endogenous jasmonic acid level, *PR* gene expression, and host resistance to fungal infection. Mol. Plant Microbe Interact..

[61] Huot B., Yao J., Montgomery B.L., He S.Y. (2014). Growth-defense tradeoffs in plants: A balancing act to optimize fitness. Mol. Plant.

[62] Campos M., Yoshida Y., Major I., de Oliveira Ferreira D., Weraduwage S.M., Froehlich J.E., Johnson B.F., Kramer D.M., Jander G., Sharkey T.D. (2016). Rewiring of jasmonate and phytochrome B signalling uncouples plant growth-defense tradeoffs. Nat. Commun..

[63] Kobayashi Y., Fukuzawa N., Hyodo A., Kim H., Mashiyama S., Ogihara T., Yoshioka H., Matsuura H., Masuta C., Matsumura T. (2020). Role of salicylic acid glucosyltransferase in balancing growth and defence for optimum plant fitness. Mol. Plant Pathol..

[64] Kong H.G., Shin T.S., Kim T.H., Ryu C.M. (2018). Stereoisomers of the bacterial volatile compound 2,3-Butanediol differently elicit systemic defense responses of pepper against multiple viruses in the field. Front. Plant Sci..

[65] Vázquez-Chimalhua E., Ruíz-Herrera L.F., Barrera-Ortiz S., Valencia-Cantero E., López-Bucio J. (2019). The bacterial volatile dimethyl-hexa-decilamine reveals an antagonistic interaction between jasmonic acid and cytokinin in controlling primary root growth of *Arabidopsis* seedlings. Protoplasma.

[66] Livak K.J., Schmittgen T.D. (2001). Analysis of relative gene expression data using real-time quantitative PCR and the 2^−ΔΔCT^ method. Methods.

