# Supplementary material for: Bacterial Compound N,N-Dimethylhexadecylamine Modulates Expression of Iron Deficiency and Defense Response Genes in Medicago truncatula Independently of the Jasmonic Acid Pathway
Source: Plants (Basel). 2020 May 14;9(5):624. doi: 10.3390/plants9050624 (PMC7285375; doi:10.3390/plants9050624)
Supplement: Supplementary file 1 [file plants-09-00624-s001.pdf]

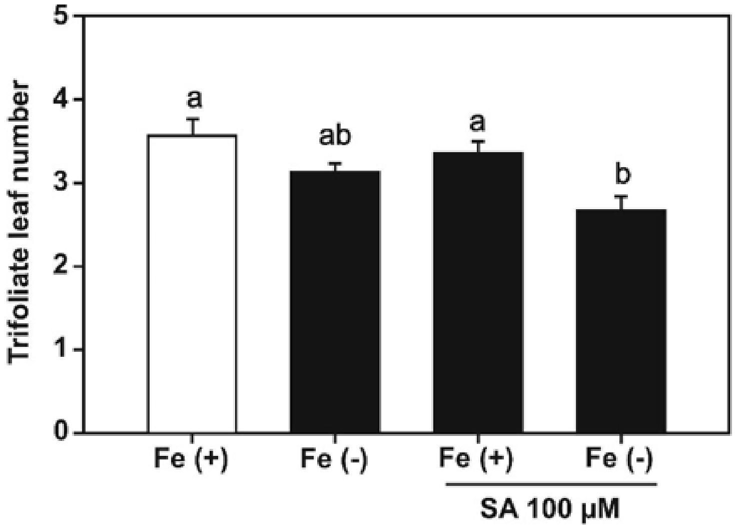

Figure S1. Effect of salicylic acid (SA) on *Medicago truncatula* trifoliate leaf number. The *M. truncatula* plants were cultured in MS medium with SA (100 µM) under both iron sufficiency (control) and iron deficiency for 14 days, after which, trifoliate leaves number were counted. Letters above the standard error bars indicate significances calculated with two way ANOVA and Tukey´s test ($p < 0.5$; $n = 9$).

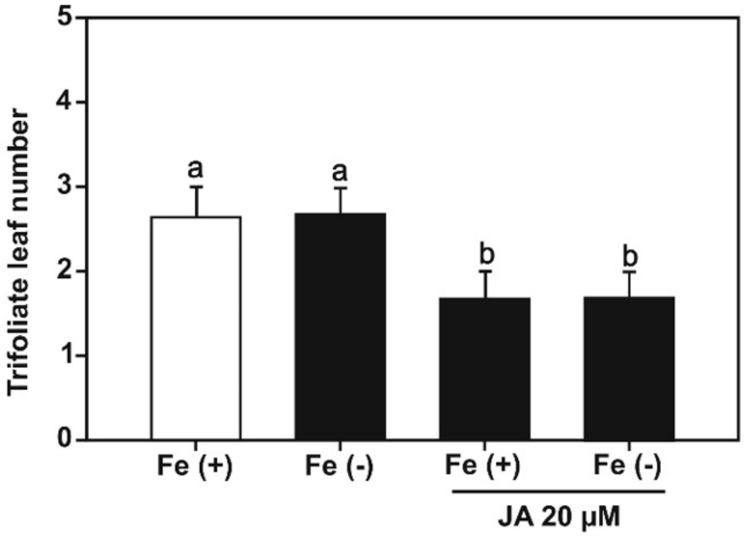

Figure S2. Figure S2. Effect of jasmonic acid (JA) on *Medicago truncatula* trifoliate leaf number. The *M. truncatula* plants were cultured in MS medium with JA (20 µM) under both iron sufficiency (control) and iron deficiency or 14 days, after which, trifoliate leaves number were counted. Letters above the standard error bars indicate significances calculated with two way ANOVA and Tukey´s test ($p < 0.5$; $n = 9$).

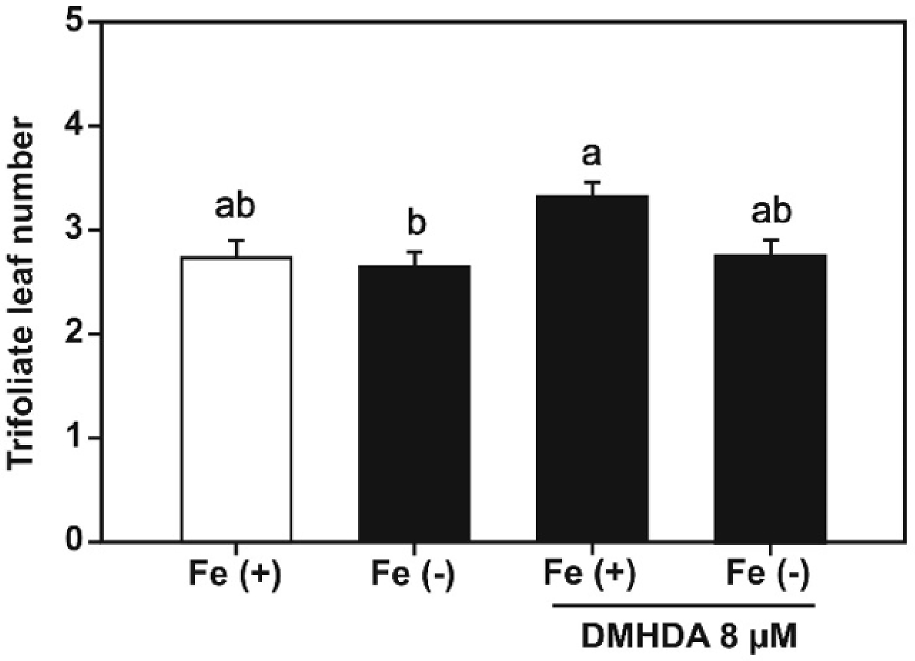

Figure S3. Effect of *N,N*-dimethylhexadecylamine (DMHDA) on *Medicago truncatula* trifoliate leaf number. The *M. truncatula* plants were cultured in MS medium with DMHDA (8 µM) under both iron sufficiency (control) and iron deficiency for 14 days, after which, trifoliate leaves number were counted. Letters above the standard error bars indicate significances calculated with two way ANOVA and Tukey´s test ($p < 0.5$; $n = 9$).

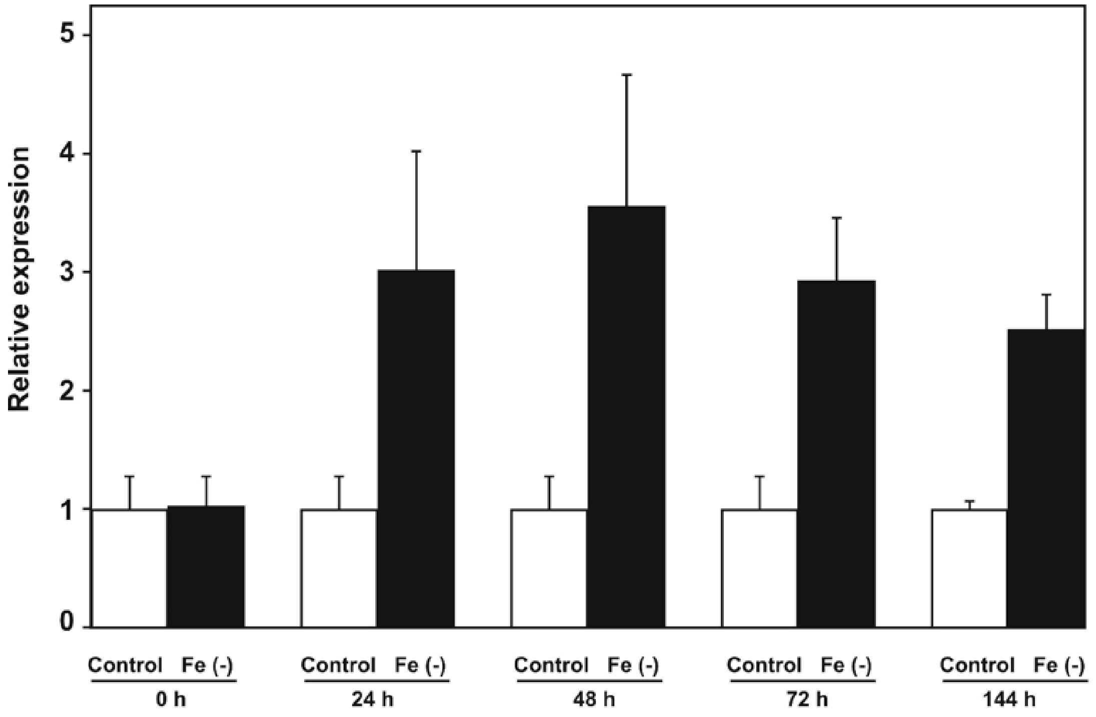

Figure S4. Figure S4. Time kinetics of *MtFIT* gene expression. The *Medicago truncatula* plants were grown in MS Figure 12. days and then transferred to MS under both iron sufficiency (control) and iron deficiency for 0, 24, 48, 72, and 144 hours, after which RNA extraction and RT-qPCR were performed. Bars represent relative expression in reference to controls and were not different according to a two way ANOVA and Tukey's test ($p < 0.5$; $n = 3$).

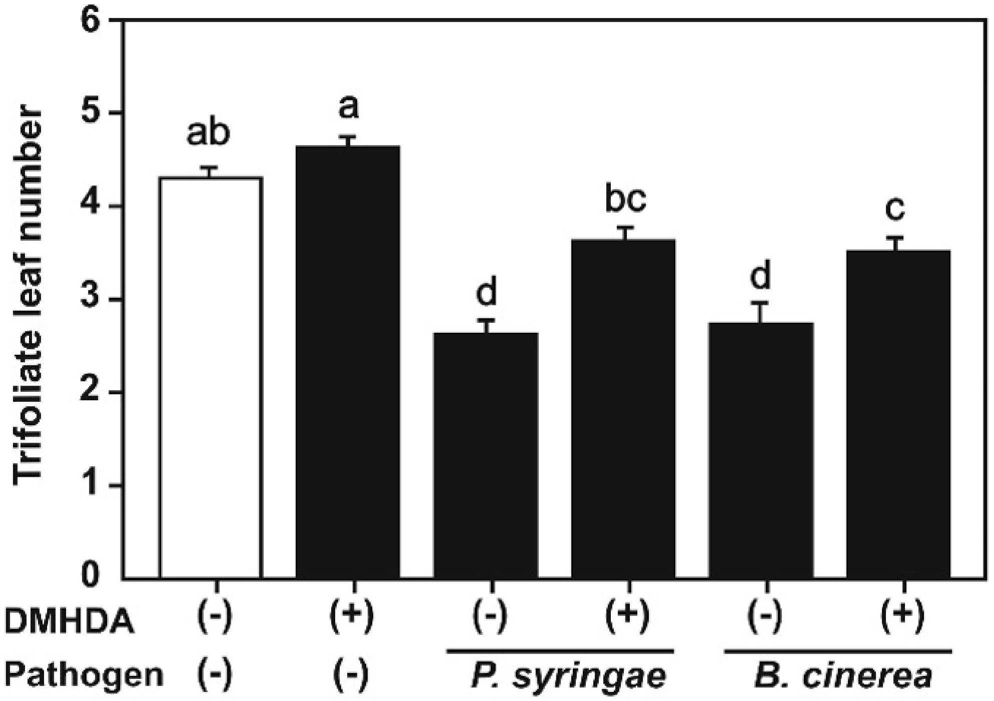

Figure S5. Effect of *N,N*-dimethylhexadecylamine (DMHDA) and *Pseudomonas syringae* or *Botrytis cinerea* infection on *Medicago truncatula* trifoliate leaf number. The *M. truncatula* plants were cultured in MS medium with (or without) DMHDA (8 μM) for five days, and then infected (or not) with *P. syringae* or *B. cinerea* and cultured for 15 days more, after which, trifoliate leaves number were counted. Letters above the standard error bars indicate significances calculated with two way ANOVA and Tukey´s test ($p < 0.5$; $n = 9$).

Fig S6. Representative images of RNA samples run on a 1% agarose gel

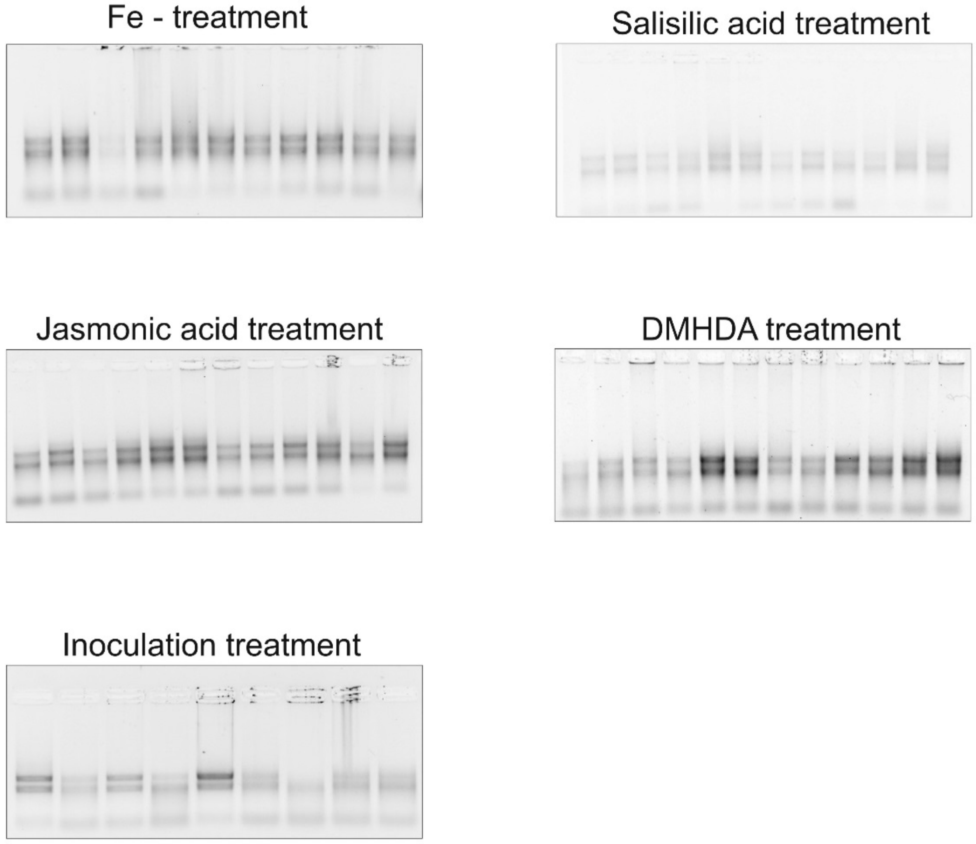

Figure S6. Representative images of RNA samples run on a 1% agarose gel.

Fig S7. Dissociation curves produced by RT-qPCR amplicons of genes listed in Table S1

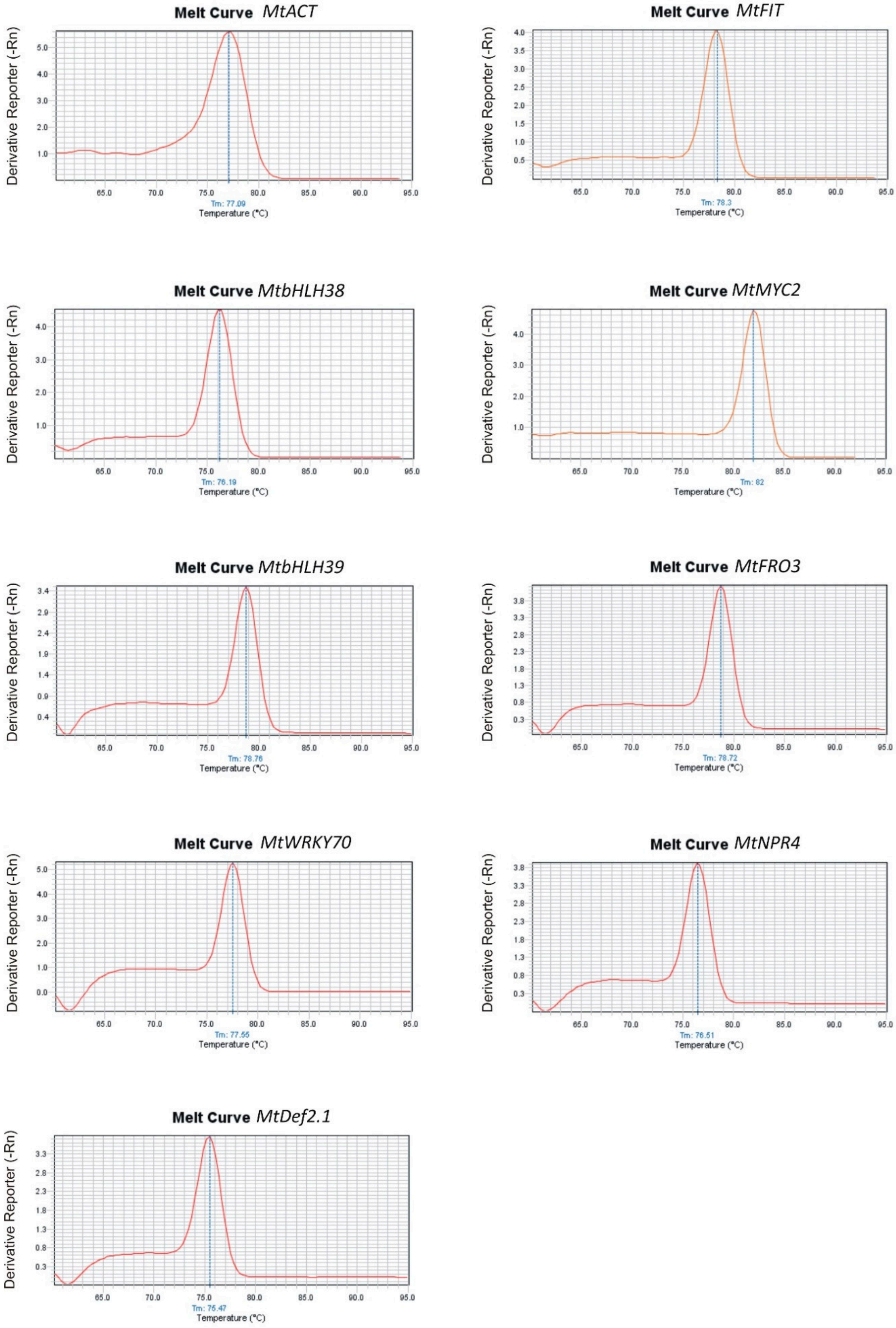

Figure S7. Dissociation curves produced by RT-qPCR amplicons of genes listed in Table S1.

Table S1. Genes identified in the present study.

Gene	CDS	Query Cover	Identity	Characteristic Domain
MtbHLH38	Medtr1g048720.1	63%	62%	Helix-loop-helix DNA-binding domain
MtbHLH39	Medtr7g090410.1	60%	60%	Helix-loop-helix DNA-binding domain
MtFIT	Medtr4g057270.1	65%	68%	Helix-loop-helix DNA-binding domain
MtNPR4	Medtr5g090770.3	77%	63%	NPR1/NIM1 like defense protein C terminal,<br>BTB/POZ domain,<br>Ankyrin repeats (3 copies)<br>Domain of unknown function (DUF3420)
MtWRKY70	Medtr3g093830.1	33%	67%	WRKY DNA-binding domain
MtMYC2	Medtr5g030430.1	55%	65%	bHLH-MYC and R2R3-MYB transcription factors N-terminal Helix-loop-helix domain

Table S2. Ratio of absorbance at 260 nm to absorbance at 280 nm of RNA samples used in the RT-qPCR measurements.

Sample ID	RNA Concentration (ng/µL)	Absorbance at 260 nm	Absorbance at 280 nm	260 nm/280 nm Absorbance Ratio	260 nm/230 nm Absorbance Ratio
RNA samples used in the experiment reported in Figure 5					
1 Fe (+)	312	7.8	3.895	2	0.97
2 Fe (+)	174.2	4.354	2.232	1.95	1.37
3 Fe (+)	196	4.9	2.501	1.96	0.95
1 Fe (−)	483.1	12.078	5.855	2.06	1.62
2 Fe (−)	371.4	9.285	4.541	2.04	1.35
3 Fe (−)	757.1	18.927	9.154	2.07	1.97
RNA samples used in the experiment reported in Figure 6					
1 Fe (+)	286.1	7.152	3.519	2.03	1.77
2 Fe (+)	217.6	5.439	2.737	1.99	1.75
3 Fe (+)	108.2	2.706	1.428	1.89	1.21
1 SA 100 µM	612.9	15.322	7.51	2.04	1.82
2 SA 100 µM	674.6	16.865	8.264	2.04	1.85
3 SA 100 µM	351.5	8.788	4.22	2.08	1.89
1 Fe (−) SA 100 µM	963.2	24.08	11.523	2.09	2.07
2 Fe (−) SA 100 µM	307	7.676	3.692	2.08	2.03
3 Fe (−) SA 100 µM	640.7	16.017	7.902	2.03	1.86
RNA samples used in the experiment reported in Figure 7					
1 Fe (+)	276.3	6.907	3.525	1.96	0.69
2 Fe (+)	313.8	7.846	3.983	1.97	0.75
3 Fe (+)	445.8	11.146	5.56	2	0.94
1 JA 20 µM	479.9	11.999	5.884	2.04	1.34
2 JA 20 µM	743.5	18.588	9.177	2.03	1.6
3 JA 20 µM	243.3	6.083	3.088	1.97	1.24
1 -Fe JA 20 µM	951.6	23.79	11.656	2.04	1.93

2 -Fe JA 20 μM	736.4	18.409	9.047	2.03	1.9
3 -Fe JA 20 μM	117	2.926	1.532	1.91	0.39
RNA samples used in the experiment reported in Figure 8					
Fe (+)	456.8	11.421	5.56	2.05	1.69
Fe (+)	620.6	15.516	7.564	2.05	1.73
Fe (+)	625.2	15.631	7.672	2.04	1.34
DMHDA 8 μM	1018.5	25.463	12.131	2.1	1.71
DMHDA 8 μM	805.4	20.134	9.769	2.06	1.48
DMHDA 8 μM	647.4	16.185	7.815	2.07	1.52
1 -Fe DMHDA 8 μM	231.9	5.798	2.909	1.99	1.45
2 -Fe DMHDA 8 μM	300	7.499	3.683	2.04	1.79
3 -Fe DMHDA 8 μM	484	12.101	5.892	2.05	1.46
RNA samples used in the experiment reported in Figure 9					
1 Fe (+)	856.3	21.406	10.293	2.08	1.55
2 Fe (+)	1113.8	27.845	13.192	2.11	1.16
3 Fe (+)	1013	25.324	12.09	2.09	2.12
1 *B. cinerea*	796	19.901	9.565	2.08	1.99
2 *B. cinerea*	601.1	15.027	7.356	2.04	1.62
3 *B. cinerea*	156.6	3.915	2.036	1.92	0.7
1 *P. syringae*	823.6	20.59	9.902	2.08	1.83
2 *P. syringae*	855.4	21.385	10.265	2.08	2.07
3 *P. syringae*	992.3	24.808	11.871	2.09	1.76

Table S3. List of oligonucleotides employed in RT-qPCR.

Gene		Nucleotide Sequence	Amplicon Size (bp)	Reference
MtACT	F	CCAATAGGGACAACAACACTTTC	209	[36]
	R	ACCAAACAGCGGATAGTAAGC		
MtbHLH38	F	CCAGCATCAGAATTCATTCTACAAA	107	This work
	R	TGCTTGTGGATTGTGAGGGA		
MtbHLH39	F	5′GCATTCTGCCCACCTCAGTT	141	This work
	R	5′TGGTGAAGAGAATTGATGATACGG		
MtFIT	F	5′GCATTGCGTTCTTTGGTTCC	124	This work
	R	5′GTCCTGCAACCTCAGCCTTA		
MtFRO3	F	AGGCGTTAGAGTGGAGCAAGAC	145	[36]
	R	GAGAATGTAGAGATGGTGAGTGTAGAAG		
MtNPR4	F	AGCATCATCATCATTGAGTTTTGTA′	112	This work
	R	TTCAGTATTTGCCATTGCCAC		
MtWRKY70	F	TGTTCTGATGGGTCTCCTTCTG	135	This work
	R	AGCTTCCACCAATGAACCTGA		
MtMYC2	F	GGCTTTCATGACCTCCTCTGATT	146	This work
	R	AGGTCCAGATTTCTTTTGCACC		
*MtDef2.1**	F	ACTTTAATACACACACCCATTTGC	125	[27,37]
	R	TCAGTTAAGATCTAGAGTCCCACA		

* *MtDef2.1* gene was identified in [37], and the nucleotides were designed in [27].
